# Supplementary material for: 3D geometry and mechanics of a single apical stem cell ensure helically symmetric plant body in multicellular models
Source: Front Plant Sci. 2026 Jul 16;17:1852286. doi: 10.3389/fpls.2026.1852286 (PMC13422487; doi:10.3389/fpls.2026.1852286)
Supplement: Supplementary file 1 [file Image1.pdf]

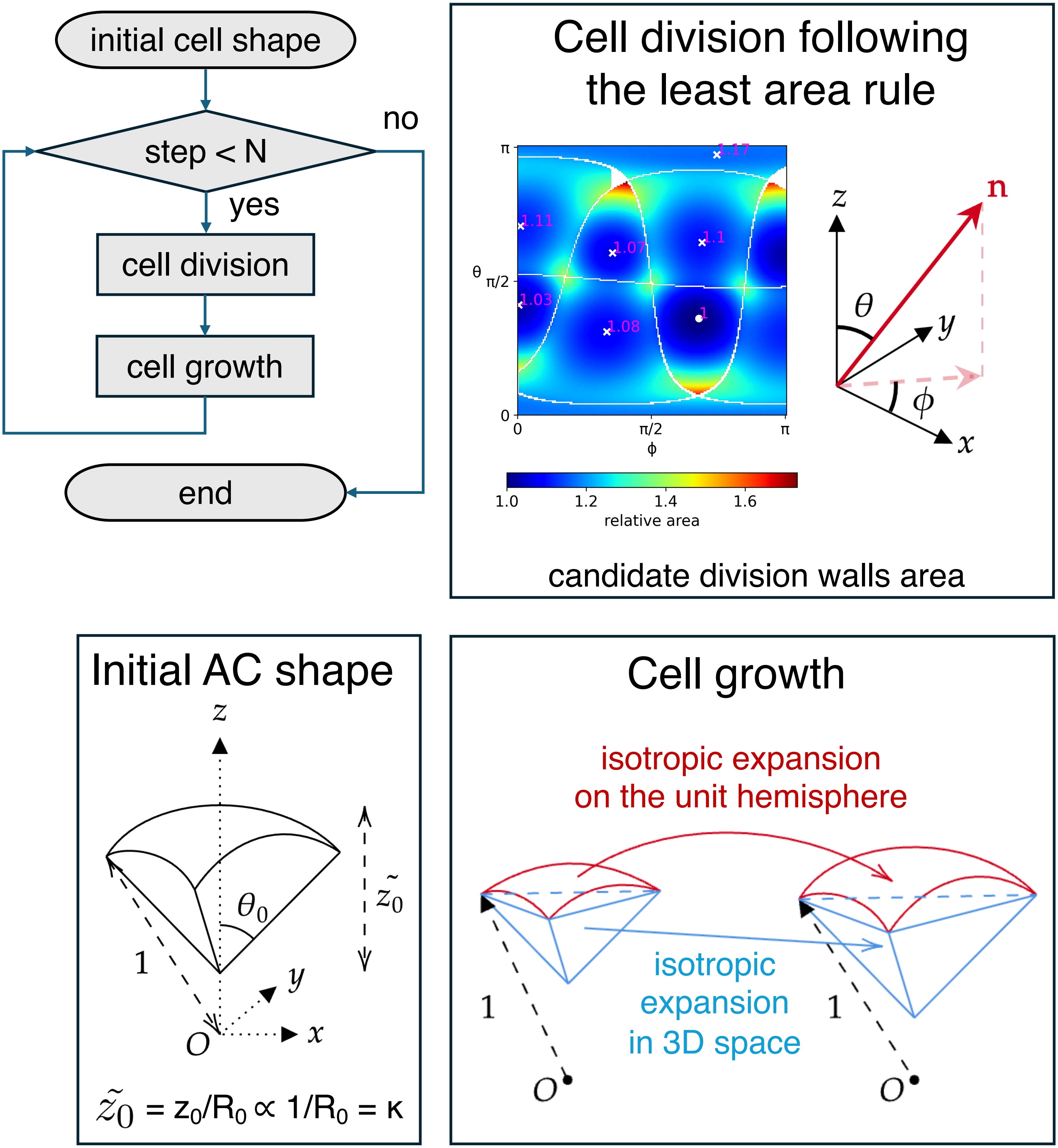

Fig. S1: **Detailed methods for the geometrical model during AC divisions.**

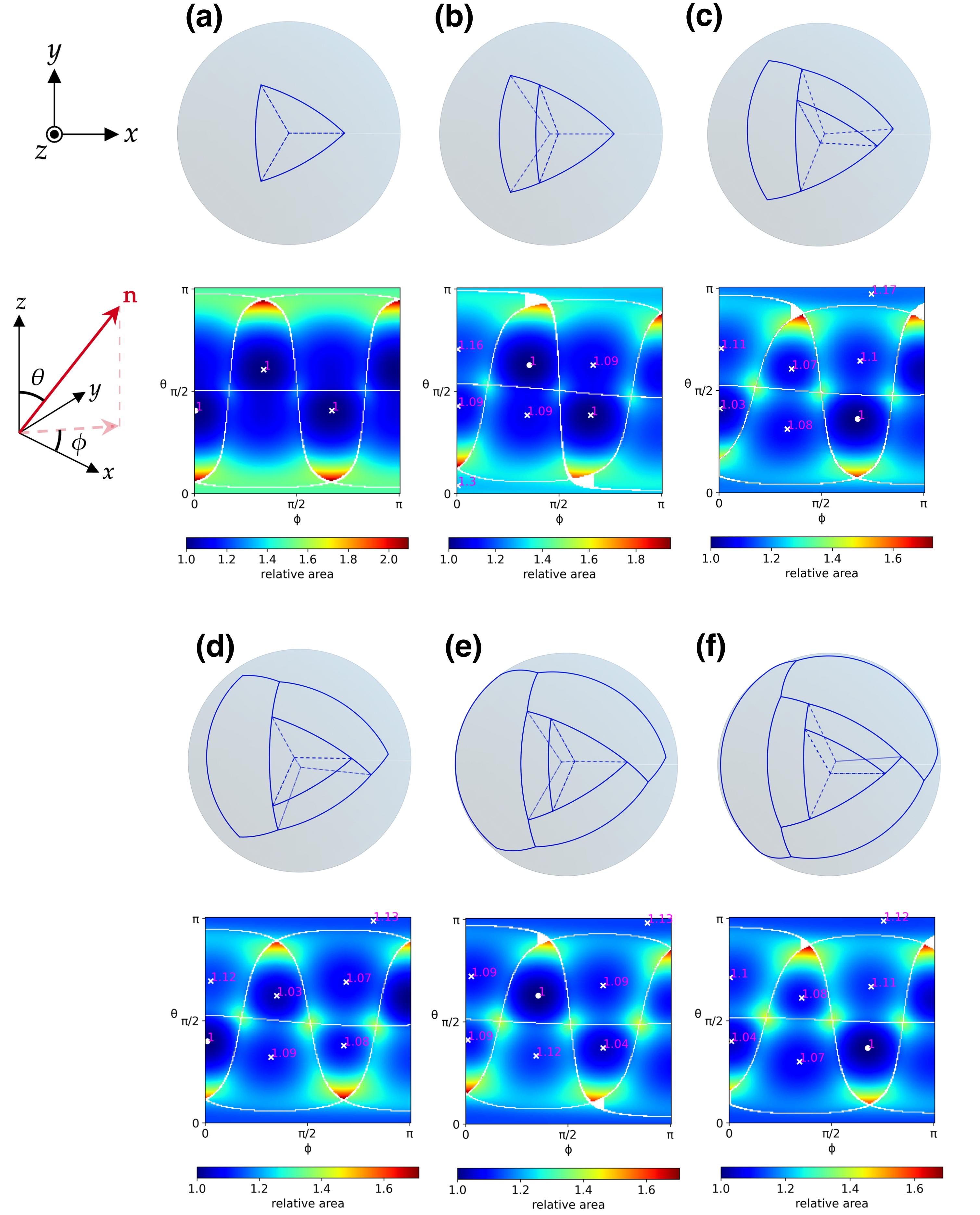

Fig. S2: **Six rounds of the least area divisions from tri-symmetric AC.** The landscape of the potential division plane area (bottom panel) passing through the centroid of the AC before the first (a), second (b), third (c), fourth (d), fifth (e) and sixth (f) divisions (top panel) in the geometrical model. $\theta_0 = 50^\circ$ and $\tilde{z}_0 = 0.5$.

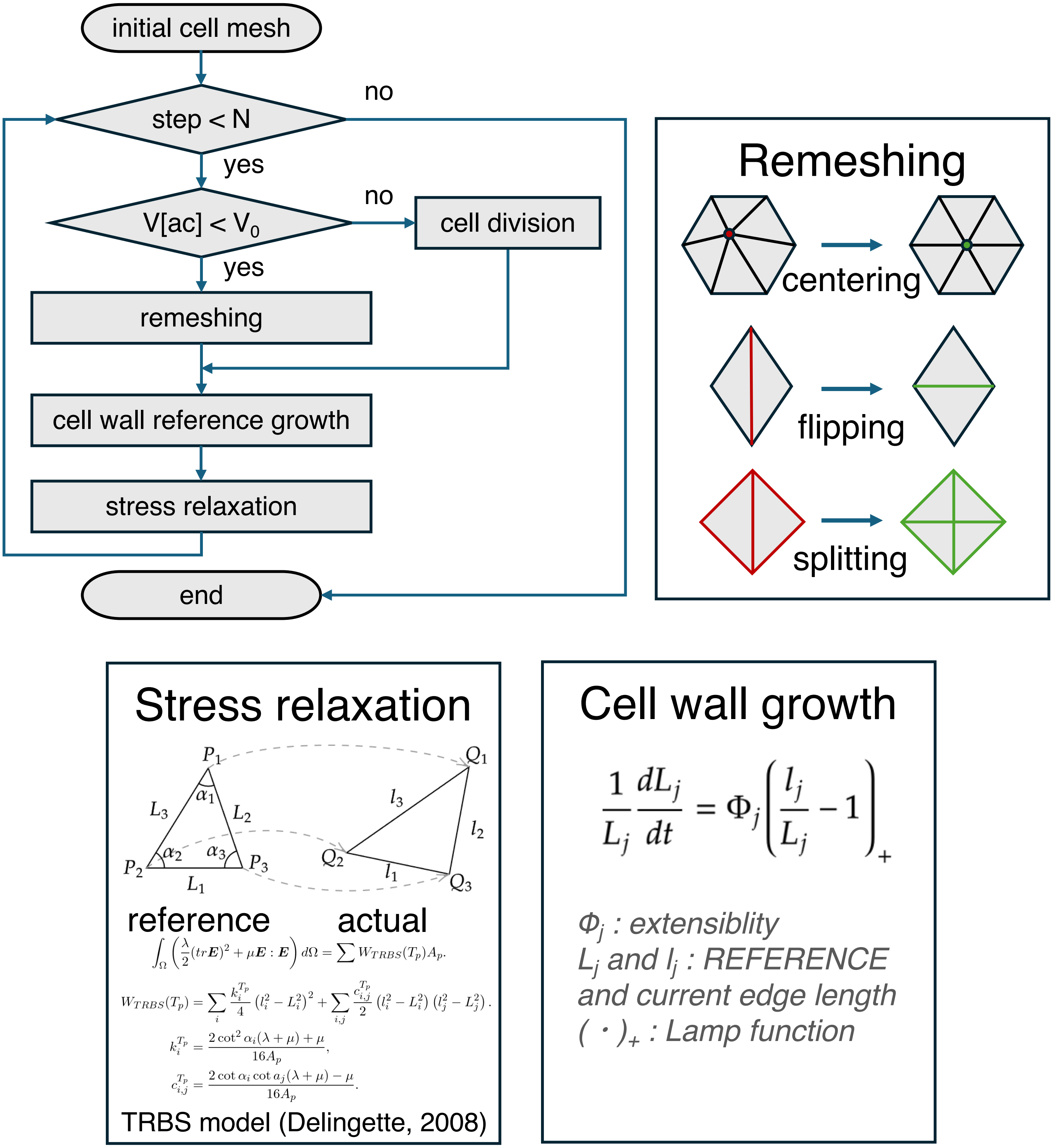

Fig. S3: **Detailed methods for the mechanical model during AC divisions.**

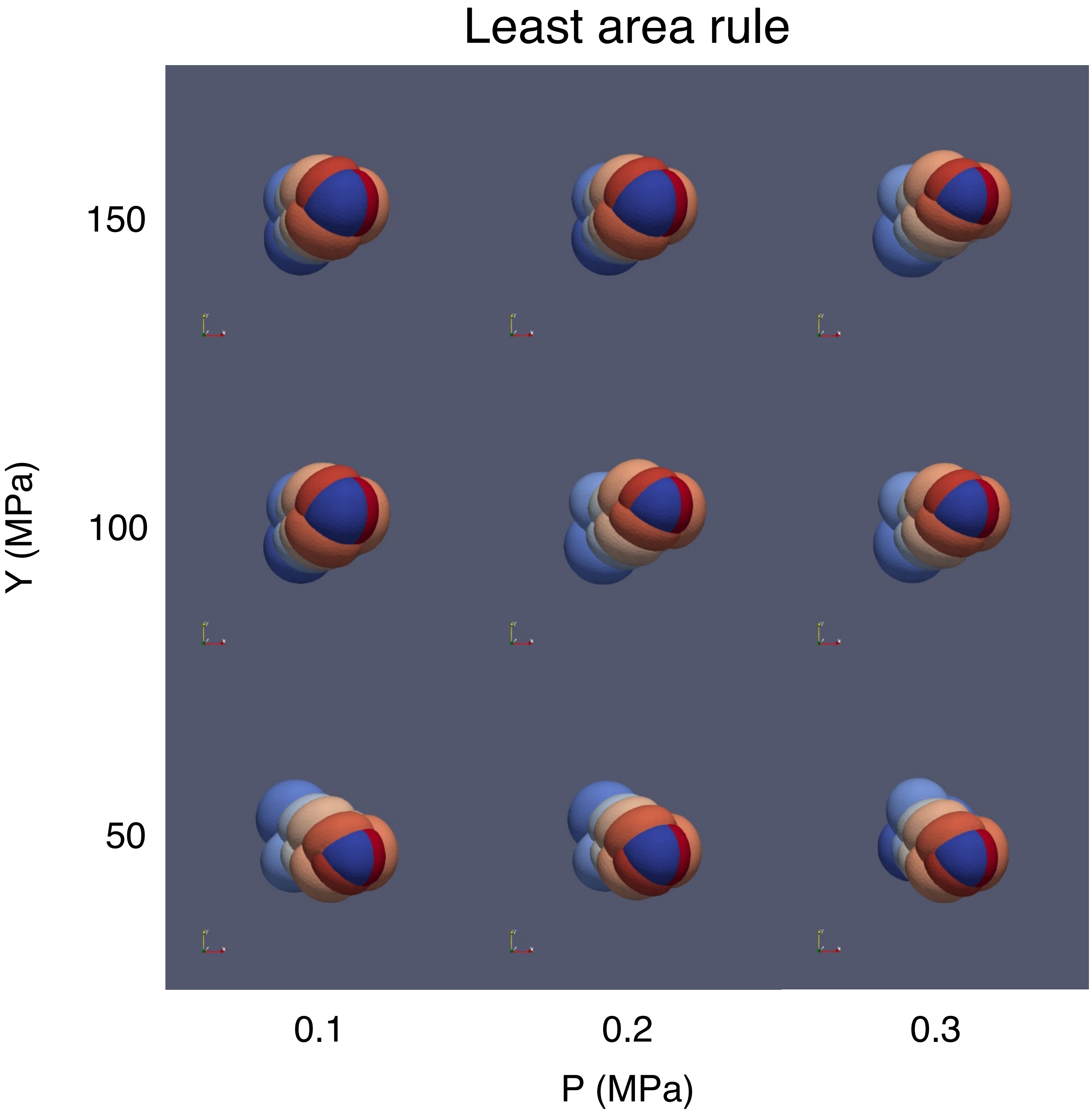

Fig. S4: **Representative temporal evolution of rotational divisions in the least area rule.** Young's modulus Y = 50 - 150 MPa by 50 MPa, Turgor pressure P = 0.1 - 0.3 MPa by 0.1 MPa. Poisson's ratio $\sigma = 0.2$.

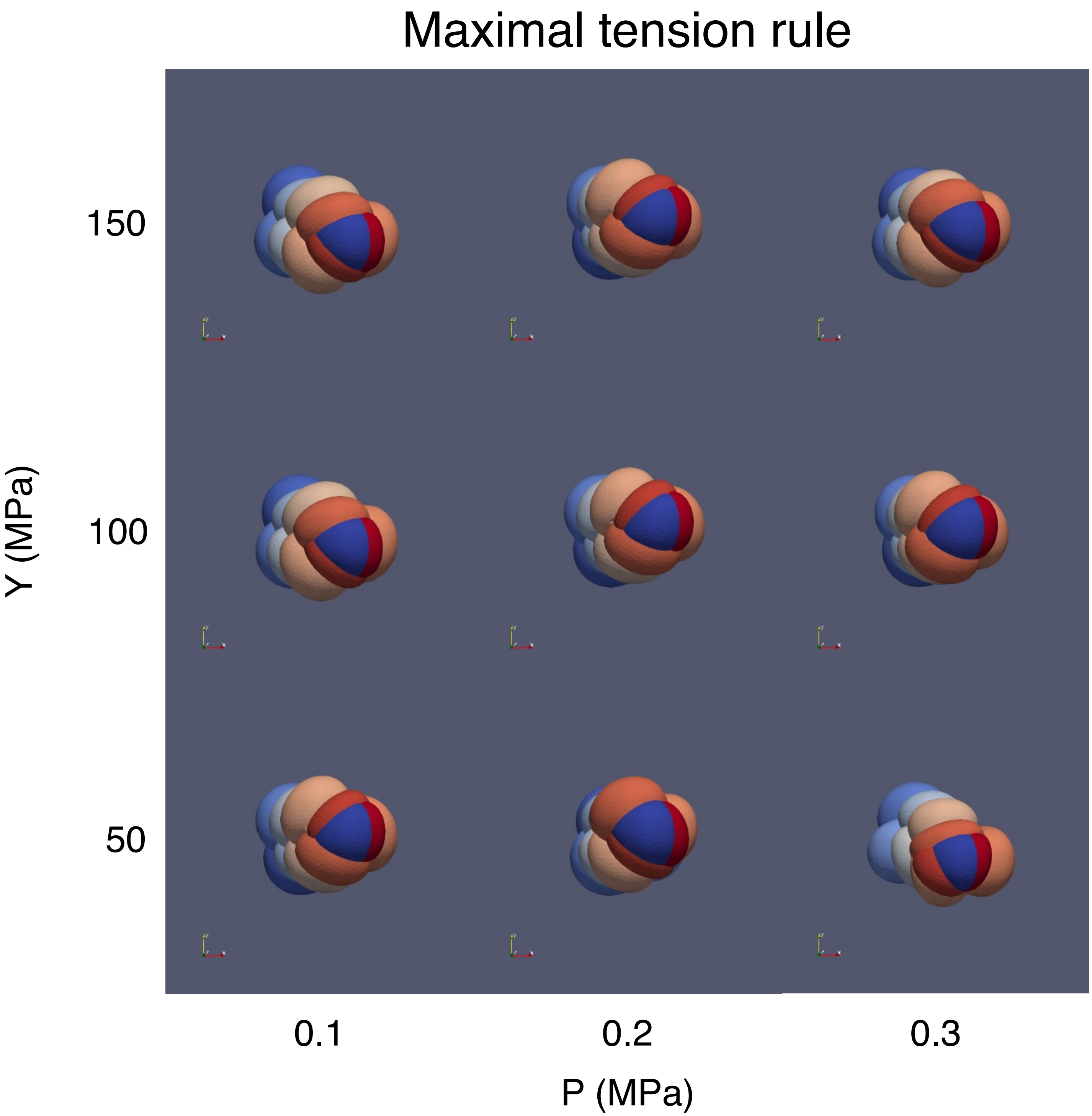

Fig. S5: **Representative temporal evolution of rotational divisions in the maximal tension rule.** Parameter range is identical to Fig. S3.4.

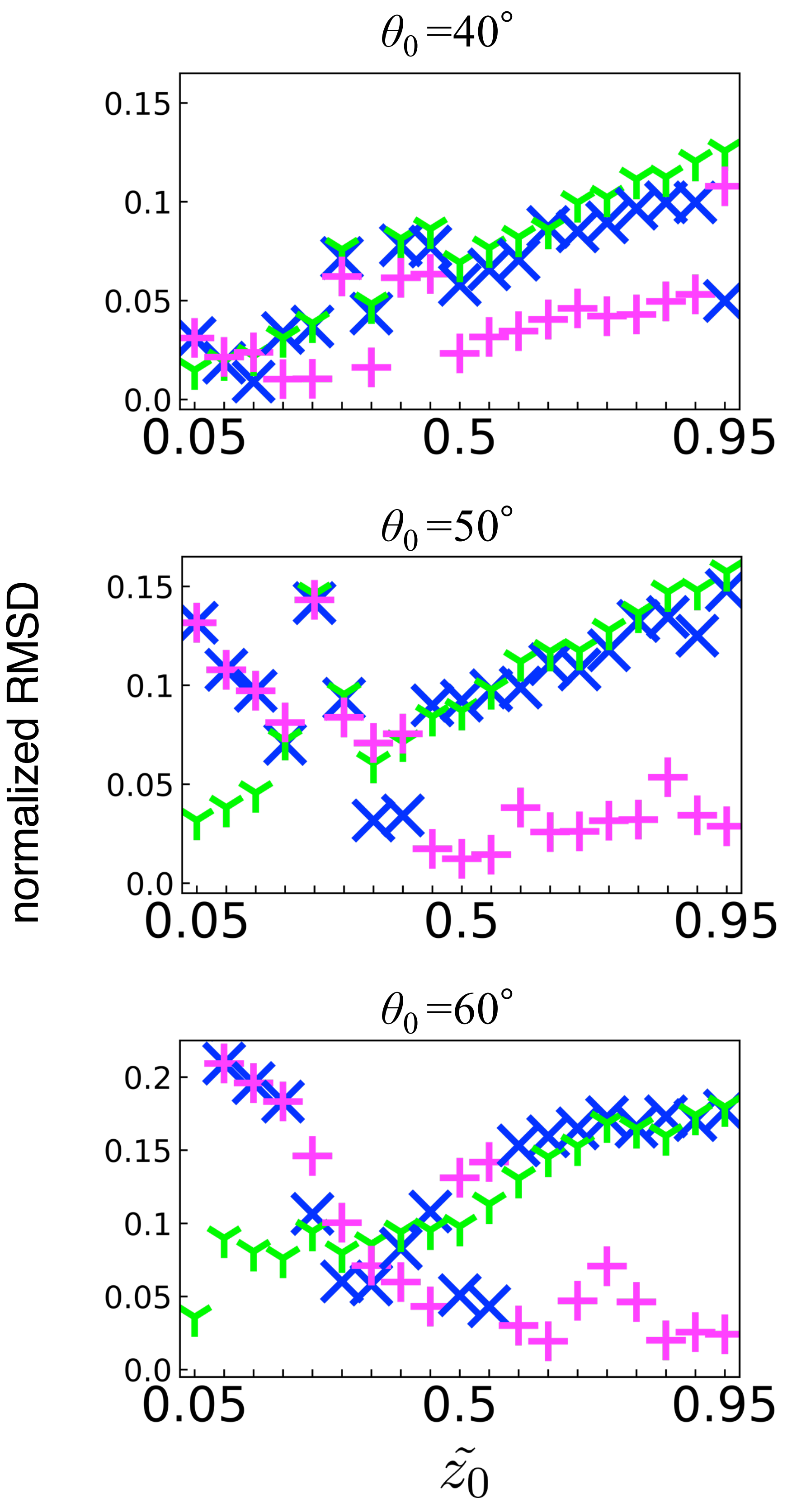

Fig. S6: **RMSD between AC outlines before and after the third cell division at various θ_0 and $\tilde{z}_0$.** Markers and colors are identical to those used in Fig. 2a.
